# Lactate and the Lactate-to-Pyruvate Molar Ratio Cannot Be Used as Independent Biomarkers for Monitoring Brain Energetic Metabolism: A Microdialysis Study in Patients with Traumatic Brain Injuries

**DOI:** 10.1371/journal.pone.0102540

**Published:** 2014-07-15

**Authors:** Juan Sahuquillo, Maria-Angels Merino, Angela Sánchez-Guerrero, Fuat Arikan, Marian Vidal-Jorge, Tamara Martínez-Valverde, Anna Rey, Marilyn Riveiro, Maria-Antonia Poca

**Affiliations:** 1 Department of Neurosurgery, Vall d’Hebron University Hospital, Universidad Autonoma de Barcelona, Barcelona, Spain; 2 Neurotraumatology and Neurosurgery Research Unit (UNINN), Vall d’Hebron University Hospital, Universidad Autonoma de Barcelona, Barcelona, Spain; 3 Neurotraumatology Intensive Care Unit, Vall d’Hebron University Hospital, Universidad Autonoma de Barcelona, Barcelona, Spain; INSERM U894, Centre de Psychiatrie et Neurosciences, Hopital Sainte-Anne and Université Paris 5, France

## Abstract

**Background:**

For decades, lactate has been considered an excellent biomarker for oxygen limitation and therefore of organ ischemia. The aim of the present study was to evaluate the frequency of increased brain lactate levels and the LP ratio (LPR) in a cohort of patients with severe or moderate traumatic brain injury (TBI) subjected to brain microdialysis monitoring to analyze the agreement between these two biomarkers and to indicate brain energy metabolism dysfunction.

**Methods:**

Forty-six patients with an admission Glasgow coma scale score of ≤13 after resuscitation admitted to a dedicated 10-bed Neurotraumatology Intensive Care Unit were included, and 5305 verified samples of good microdialysis data were analyzed.

**Results:**

Lactate levels were above 2.5 mmol/L in 56.9% of the samples. The relationships between lactate and the LPR could not be adequately modeled by any linear or non-linear model. Neither Cohen’s kappa nor Gwet’s statistic showed an acceptable agreement between both biomarkers to classify the samples in regard to normal or abnormal metabolism. The dataset was divided into four patterns defined by the lactate concentrations and the LPR. A potential interpretation for these patterns is suggested and discussed. Pattern 4 (low pyruvate levels) was found in 10.7% of the samples and was characterized by a significantly low concentration of brain glucose compared with the other groups.

**Conclusions:**

Our study shows that metabolic abnormalities are frequent in the macroscopically normal brain in patients with traumatic brain injuries and a very poor agreement between lactate and the LPR when classifying metabolism. The concentration of lactate in the dialysates must be interpreted while taking into consideration the LPR to distinguish between anaerobic metabolism and aerobic hyperglycolysis.

## Introduction

High intracranial pressure (ICP) and brain ischemia have traditionally been considered the main secondary intracranial insults, and have thus been the subject of most traumatic brain injury (TBI) research. However, evidence accumulated in the last decade indicates that ischemia (ischemic brain hypoxia) is the least frequent etiologic factor in TBI-related energetic disturbances [1], [2]. Several studies have shown that disturbances in energy metabolism pathways are frequent in experimental models and clinical studies of TBI. A better understanding of the metabolic events that occur in the acute and subacute phases of TBI is essential for detecting these abnormalities at the bedside and to be better able to reverse or modulate such changes. Microdialysis (MD) probes, alone or in combination with brain oxygen monitoring, allow for the direct assessment of brain energetic metabolism via the detection of quasi-real-time changes in the concentration of the brain’s energetic substrate (i.e., glucose), the intermediate product of glycolysis (i.e., pyruvate) and the product of both aerobic and anaerobic metabolism (i.e., lactate).

For decades, lactate has been considered an excellent biomarker for oxygen limitation and therefore of organ ischemia; as a result lactate is often regarded as an essential biomarker for managing circulatory failure. Most clinical research in hypoperfused organs, including the brain, is based on the “anaerobic threshold” concept introduced by Wasserman and McIlroy in 1964 [3]. According to the traditional paradigm, increase in lactate in any organ or in blood, even during muscular contraction or exercise, is a direct consequence of tissue hypoxia. Under conditions of oxygen limitation, the inhibition of the respiratory chain increases the mitochondrial and cytosolic pools of reduced nicotinamide adenine dinucleotide (NADH) and as a consequence lactate production increases [4].

In accordance with conventional theory, in patients with acute brain injuries, an increase in brain extracellular lactate levels above a somewhat arbitrary and variable threshold (i.e., 2.0–4.0 mmol/L) has been considered an indicator of increased anaerobic glycolysis and brain hypoxia [1], [5]–[8]. However, the lactate-to-pyruvate ratio (LPR) is considered a more robust indicator of anaerobic metabolism and the redox status of the tissue, and has been found to be an independent predictor of mortality and unfavourable outcome in a multivariate analysis of the largest cohort of TBI patients monitored with MD [7]. In TBI clinical research, lactate and the LPR have been used as equivalent indicators of ischemic and non-ischemic brain hypoxia. This has confounded the discussion regarding brain metabolism impairment and the potential benefit of some therapies. Normobaric hyperoxia could be used as a potential treatment to improve brain oxygenation and consequently the metabolic disorders resulting from TBI. However, contradictory results have raised significant controversy regarding this treatment, in part due to the diverse metabolic criteria used to evaluate the brain’s response to the hyperoxic challenge, mostly based in brain lactate levels without taking into consideration changes in pyruvate and in the LPR [8]–[13].

The conventional interpretation of high brain lactate levels was first challenged by the studies of Vespa et al. which showed that in TBI patients, increases in lactate may indicate hyperglycolysis or metabolic crisis and not necessarily ischemia [2]. These studies in the acute brain injuries aligns with subsequent evidence indicating that lactate is a non-specific biomarker of an increased glycolytic flux, but that the increase in glycolysis can have multiple etiologic factors other than tissue hypoxia [14]. The resting brain releases a small amount of lactate that increases by 3- to 4-fold during brain activation and there is growing evidence that lactate may be a fuel used by neurons under aerobic conditions in agreement with the hypothesis of the astrocyte-neuron lactate shuttle suggested by Pellerin and Magistretti [15]. For a comprehensive review of the recent controversies regarding theory of lactate as a fuel and signaling molecule we recommend the recently published Dienel’s review [16].

In a pilot study published by our group we found a high prevalence of increased lactate and a striking discordance between the concentration of lactate and the LPR [17]. The present study aimed to evaluate the frequency of increased brain lactate levels and the LPR in a larger cohort of TBI patients and to analyze the agreement between these two biomarkers, thus determining whether they could be used independently to indicate brain energy metabolism dysfunction.

## Materials and Methods

### Subject selection and study design

Between September 1999 and March 2013, 188 patients with moderate/severe TBI were monitored using brain MD. Moderate/severe TBI was defined as an admission Glasgow coma scale score ≤13 after resuscitation and in the absence of paralytic agents or sedation. The Traumatic Coma Data Bank (TCDB) classification [18] was used to stratify patients. All patients were admitted to a dedicated 10-bed Neurotraumatology Intensive Care Unit (NICU) at Vall d’Hebron University Hospital, a tertiary center dedicated to the management of acute traumatic brain injuries. For all patients, clinical information, such as ICP monitoring data and systemic data, which were collected at least hourly, was stored in an in-house specially dedicated database in which demographic, monitoring and outcome variables are routinely recorded by research fellows upon discharge of patients from the NICU. All patients were treated according to the Brain Trauma Foundation guidelines for adult TBI patients, which were incorporated into our routine management protocols in 1996 [19], [20].

A randomly generated list of numbers was used to select a random sample of 54 patients from this cohort. This strategy allowed us to confirm the quality of the neuromonitoring data and review the MD data; we could thus verify that patients had at least 12 hours of complete monitoring, reject artifacts and verify the correct placement of the MD probe. To avoid variability in selection criteria, a single person (MAM) was responsible for conducting this analysis. Of the selected 54 patients, 8 were excluded because data concerning energetic metabolites (i.e., lactate, pyruvate and glucose) were missing. The remaining 46 patients with complete metabolic data were included in this study.

This cohort yielded 6530 samples. After verification, the data set was reduced to 5305 samples (81% of the total samples) that had good quality data, in which predefined artifacts and doubtful measurements were excluded. Six months after injury, outcomes were assessed by an independent neuropsychologist using the Extended Glasgow Outcome Scale (GOSE). The obtained scores were sorted into two categories: bad outcome (i.e., GOSE: 1–4) and good outcome (i.e., GOSE: 5–8).

### Ethics statement

This study received institutional approval (protocol number PI-030153). The Institutional Review Board waived the need for informed consent because MD is routinely performed in all TBI patients admitted to our Neurotraumatology Intensive Care Unit.

### ICP monitoring and general management

Continuous ICP monitoring was performed in all patients using a Camino Model 110-4B intraparenchymatous ICP sensor (Integra Neurosciences, Plainsboro, NJ, USA). Our complete ICP monitoring protocol in neurocritical patients has been previously published [21]. Due to the significant interhemispheric ICP gradients in patients with focal lesions [22], the ICP sensor was always implanted in the “worst” hemisphere, which was defined as the hemisphere with the most evident lesion, in the pre-coronal region, 11 cm from the nasion and 3 cm from the midline. End-hour ICP readings were recorded manually by patient nurses. All patients received standard treatment that aimed to maintain the following therapeutic targets: a cerebral perfusion pressure above 60 mmHg and an ICP below 20 mmHg. No clinical decisions were based on MD data, except for decisions to screen for potential secondary intra- or extracranial insults that may induce significant changes in MD trends.

### Brain microdialysis monitoring

Most patients who require ICP monitoring and lack contraindications for multimodality neuromonitoring are routinely monitored using MD probes. Brain microdialysis catheters are inserted into non-injured brain tissue according to a previously described methodology [23]. A CMA-70 brain microdialysis catheter with a 20-kDa cut-off membrane (MDialysis AB, Stockholm, Sweden) was used in 16 patients, and a CMA-71 catheter with a 100-kDa cut-off membrane (MDialysis AB, Stockholm, Sweden) was used in 30 patients. The position of the catheter was confirmed by a control CT scan in which the gold-tip of the catheter is always visible. In addition, all patients were monitored using a CMA-60 microdialysis catheter (MDialysis AB, Stockholm, Sweden) inserted into the subcutaneous adipose tissue of the abdominal region, and data was collected hourly and used as a systemic reference.

Cerebral catheters were perfused with sterile isotonic fluid containing 147 mmol/L NaCl, 1.2 mmol/L CaCl_2_, 2.7 mmol/L KCl, and 0.85 mmol/L MgCl_2_ at a fixed flow rate of 0.30 µL/min using a CMA-106 pump (MDialysis AB, Stockholm, Sweden), and the dialysates were collected in capped microvials specially designed to collect micro-volumetric samples and minimize evaporation. The microdialysate samples were collected hourly by patient nurses.

For the purpose of the analysis, data from the first two samples were always excluded. Lactate, pyruvate and glucose levels were routinely monitored using a CMA-600 microdialysis analyzer (MDialysis AB, Stockholm, Sweden), with glutamate, urea, or glycerol chosen as the fourth analyte, depending on the patient. The CMA detection intervals, as provided by the manufacturer, are 0.1–25 mmol/L for glucose, 0.1–12 mmol/L for lactate, and 10–1500 µmol/L for pyruvate. Concentrations below the level of detection were replaced by a value corresponding to the lower level of detection of the analyte divided by two, as suggested by other authors [24], [25]. The microvials were placed in a microvial rack after analysis, which was designed to seal the microvials and prevent evaporation (MDialysis AB, Stockholm, Sweden). The microvial rack was stored at 4°C at the bedside before transfer to −80°C for long-term storage.

### Metabolic thresholds

The range of normality for brain lactate, the LP ratio and the “anaerobic” threshold is still quite arbitrary and usually extrapolated from animal studies, studies in other organs or from brain MD conducted in patients who underwent neurosurgical operations for posterior fossa lesions or epilepsy [5], [26], [27]. The best estimate we have for the upper lactate and LPR thresholds are from studies of CSF and from a few studies conducted in patients in whom brain MD were monitored under general anesthesia (using different anesthetic management and/or operated on neurosurgical procedures). In these studies the upper range of normality in normal brain tissue for lactate is extremely variable and ranges from 1.50 to 5.10 [7], [14], [27]. A recent study for the reference ranges in the CSF established an upper 95% percentile at 2.6 mmol/L [28]. Reinstrup et al. measured energetic metabolism in nine awake and anesthetized patients using the same probes (10 mm length and 20 kDa cut-off) and flow rate that were used in our study (0.3 µL/min), and that are used by most groups in the clinical setting [5].

Perfusion speed is known to influence the relative recovery of any extracelular fluid (ECF) metabolite, whereas the LPR remains stable and is less prone to be affected by methodological issues. In most studies that have provided reference ranges the LPR has a more consistent upper threshold ranging from 15 to 25 [7], [29]. For the purpose of this study we selected a 2.5 mmol/L threshold for lactate (corresponding to an actual brain tissue lactate of around 3.0 mmol/L) and a LPR of 25. To compare our data with recently published studies we also conducted the analysis with the proposed thresholds for lactate of 4.0 mmol/L [6], [7].

### Statistical analysis

Descriptive statistics were obtained for each variable. For continuous variables, summary statistics were the mean, median, range and the standard deviation. Percentages and sample sizes were used to summarize categorical variables. The Shapiro-Wilks test and inverse probability plot were used to test whether data followed a normal distribution. To correlate two continuous variables the most conservative Kendall’s tau (when data did not follow a normal distribution) or Pearson correlation test (for data following a normal distribution) was used. To model the dependence of the LPR on lactate simple linear regression and the Ordinary Least Squares (OLS) method were used with lactate as the independent variable. Adjusted R^2^ were calculated for all models to test whether linear or non-linear models adequately explained the relationships between both variables.

Statistical analyses were carried out with R (version 3.0.1) [30] and the integrated development environment R Studio (v0.97.551) [31]. The car package was used for regression analysis [32]. For evaluating agreement between lactate and the LP ratio the overall proportion of agreement, the unweigthed Cohen’s Kappa and Gwet’s AC1 [33] were used to calculate chance-corrected inter-rater reliability coefficients using AgreeStat v2011.3 (Advanced Analytics, Gaithersburg, MD, USA). Gwet’s AC1 statistic is more robust than Cohen’s Kappa in avoiding what has been called the “Kappa paradox”, the situation in which Kappa values are low despite a high percentage of agreement [33]. We established a Kappa and Gwet’s AC1 of at least 0.61 as the minimum acceptable degree of agreement to consider both lactate and the LPR as interchangeable in the clinical setting [34].

## Results

Forty-six (36 men and 10 women) patients with a median initial Glasgow coma sale score of 6 were included in the study. Mean age of the group was 35±15 years. We included 26 patients with a diffuse type II injury, 5 patients with a diffuse type III injury, 5 patients with a diffuse type IV injury, 8 patients with an evacuated mass lesion (V) and 2 patients with a non-evacuated mass lesion (VI). Demographic and clinical characteristics of this cohort are summarized in **Table 1**. Because of the changes in the CT scan pattern, we verified that the MD probe was always placed in a macroscopically normal brain in all patients. Six-month mortality rate was 17.4% (8 patients). Five patients could not be contacted for long-term follow-up and thus it was not possible to assess the six-month GOSE. Of the remaining 33 patients, 15 presented a good outcome and 18 a bad outcome.

**Table 1:** Demographic and clinical characteristics of the patients.

Sex^a^		
Man	36	(78%)
Woman	10	(22%)
Age^b^	35	(±15)
Initial GCS^c^	6	(5,8)
Initial CT classification^a^		
II	26	(56.5%)
III	5	(10.9%)
IV	5	(10.9%)
V	8	(17.4%)
VI	2	(4.3%)
GOSE (6 months)^a^		
Good outcome	15	(32.6%)
Bad outcome^d^	18	(39.1%)
Dead^e^	8	(17.4%)
Lost to 6-months follow-up	5	(10.9%)

GOSE, Extended Glasgow Outcome Scale; GCS, Glasgow.

Coma Scale.

a Number of cases (percentage).

b Mean (standard deviation).

c Median (first quartile, third quartile).

d Patients with upper or lower severe disability or vegetative state.

e Mortality at hospital discharge.

### Lactate, pyruvate and glucose extracellular concentrations

**Figure 1:**
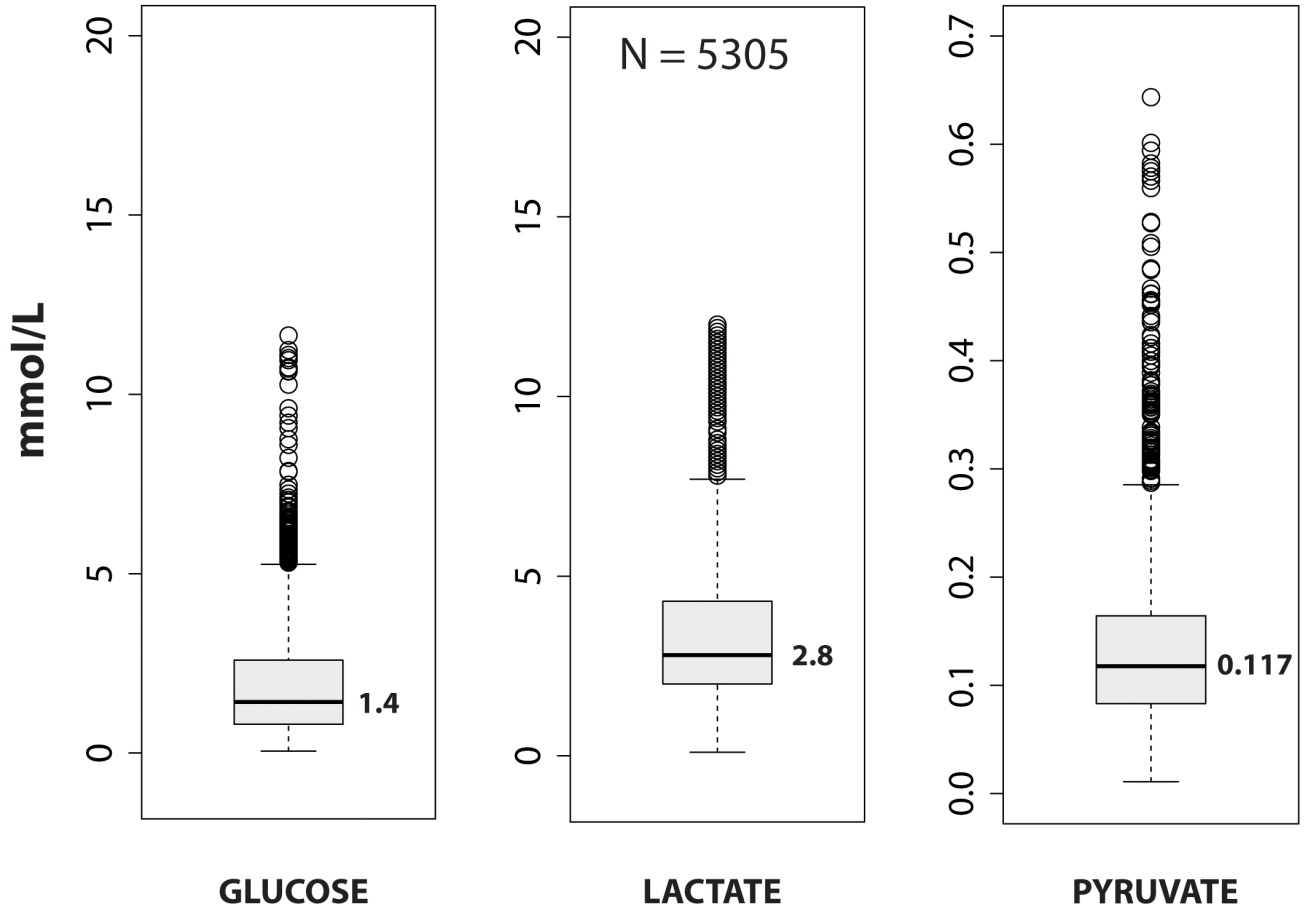
Box-and-whisker plots summarizing the distribution of data for glucose, lactate, and pyruvate. The rectangular box spans the first to the third quartile of data and the median, marked in the center of the box, is labeled with its value to the right. Whiskers extend to the furthest observations within ±150% of the interquartile range. Observations outside 150% of the interquartile range are marked as outliers with dots. N = 5305 represents all valid matched data for the three variables. All variables were highly skewed to the right. For an explanation, see the text in the results section.

The 46-patient group yielded 5305 verified samples of good data in which predefined artefacts and uncertain or incomplete measurements had been excluded. **Figure 1** presents a summary of the glucose, lactate and pyruvate data. None of the variables were normally-distributed and were highly skewed to the right. The median for glucose was 1.42 (min: 0.05, max: 11.64), 2.80 for lactate (min: 0.10, max: 12.00) and 0.117 for pyruvate (min: 0.011, max: 0.644), all in mmol/L. The median for the LPR was 23.9 (min: 2.0, max: 545.5). In 28 microvials (0.53%) LP ratio was above 150. In all cases, the extreme LPR values were due to a remarkably low pyruvate (0.01 to 0.06 mmol/L). Lactate was above 2.5 mmol/L in 3018 readings (56.9%) (**Table 2**). If the lactate threshold was increased to 3.0 mmol/L, 2457 determinations (46.3%) were above this threshold, while 1496 (28.2%) would be above 4.0 mmol/L threshold. The LPR was above the predefined threshold of 25 in 2309 determinations (43.5%).

**Table 2:** Intermethod agreement in the classification of dialysate samples by lactate and the LPR.

				*Lactate*		
				*Normal metabolism*	*Abnormal metabolism*	*Total*
				*L ≤2.5*	*L >2.5*	
***Lactate-to-pyruvate ratio***	***Normal metabolism***		***N***	1722	1274	2996
	*LP ratio ≤25*		***%***	32.5%	24.0%	56.5%
	***Abnormal metabolism***		***N***	565	1744	2309
	*LP ratio >25*		***%***	10.7%	32.9%	43.5%
	***TOTAL***		***N***	2287	3018	**5305**
			***%***	43.1%	56.9%	**100%**

LP, lactate-to-pyruvate; L, lactate; >, greater than; ≤, less than or equal to; N, number of readings; %, percentage.

### Correlation between lactate and the LP ratio

**Figure 2:**
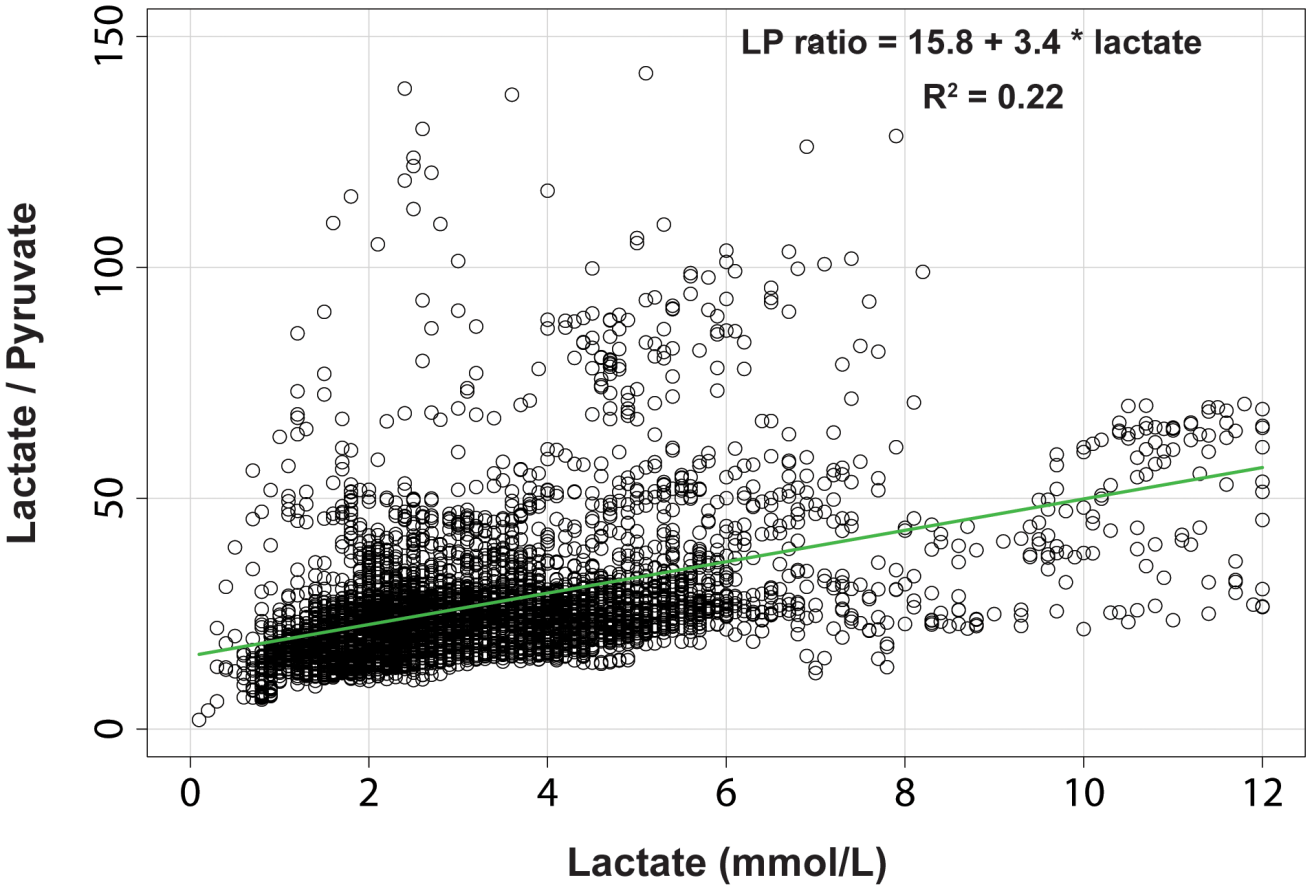
Scatter plot of the relationship between lactate and the lactate-to-pyruvate ratio. The best-fit straight line using an ordinary least squares method is included. In this plot, outliers with a lactate-to-pyruvate ratio >150 were excluded. The plotted residuals and other regression diagnostics show that the relationships between lactate and the lactate-to-pyruvate ratio could not be adequately modeled by any linear or non-linear model.

A scatter plot of these two variables is shown in **Figure 2**. When all the data were considered, no linear relationship between lactate and the LP ratio was found (adjusted R^2^ = 0.15). When all outliers with an LP ratio >150 were excluded (28 samples, 0.53%), a very modest linear regression model was fit and the variables were found to be correlated (adjusted R^2^ = 0.22). However, the plotted residuals showed that the relationships between lactate and the LP ratio could not be adequately modeled by any linear or non-linear model. This fact suggests that the variations in pyruvate were very influential but that they could not be used as an independent predictor of the LPR (data not shown). The same analysis was conducted between lactate (predictor) and the lactate-to-glucose ratio (outcome), but we could not fit a linear or non-linear model to these data either (R^2^ = 0.03).

### Agreement between lactate and the LP ratio

To calculate the agreement between lactate and the LPR in classifying hourly microdialysates, we assumed that all 5305 readings were rated independently by the two biomarkers where the ratings were based on a dichotomous scale consisting of 1) normal metabolism or 2) abnormal metabolism. The thresholds of 2.5 mmol/L for lactate and 25 for the LP ratio were used to sort the patients. From the generated contingency tables **(** **Table 2** **)**, the overall proportion of agreement, the un-weighted Cohen’s kappa, and Gwet’s AC1 statistic were used to calculate chance-corrected inter-rater reliability coefficients. The percentage of agreement was 65%, however Cohen’s kappa was 0.32 (95% CI: 0.29–0.34) and Gwet’s AC1 was 0.31 (95% CI: 0.28–0.33). None of the statistics showed an acceptable chance-corrected agreement in classifying patients with normal or abnormal metabolisms. When using a contingency table with a higher threshold for lactate (4.0 mmol/L) and the value of 25 for the LPR used by other authors [6], [7], neither Cohen’s kappa (0.27, 95% CI: 0.25–0.30) nor Gwet’s AC1 (0.36, 95% CI: 0.34–0.39) were above what is considered moderate agreement [34].

### Metabolic patterns

**Figure 3:**
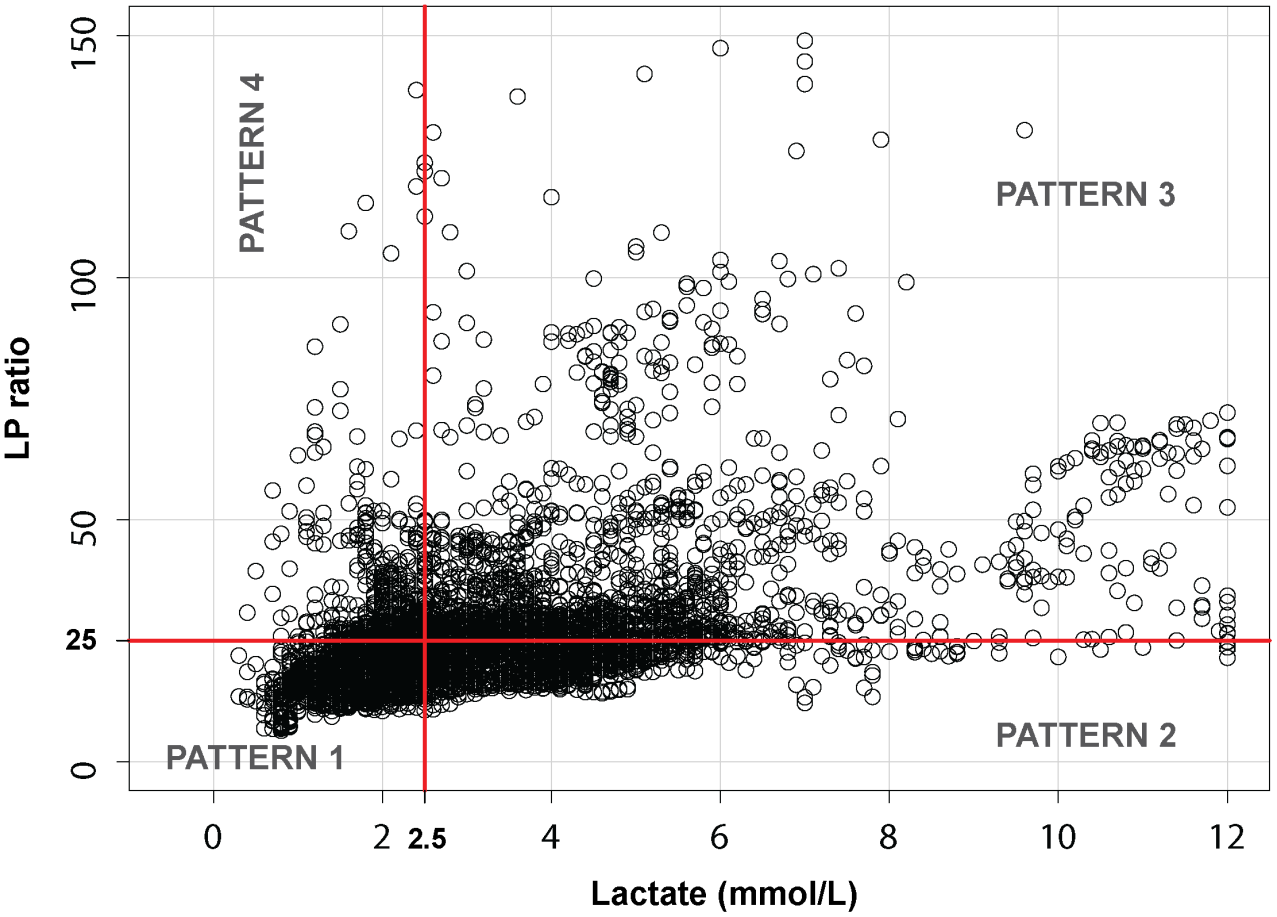
Scatter plot for lactate and the lactate-to-pyruvate ratio. The entire dataset is divided in the four patterns described in the text. We propose the following terminology for the four patterns: Pattern 1 (normal metabolism), Pattern 2 (aerobic hyperglycolysis), Pattern 3 (anaerobic metabolism), and Pattern 4 (low pyruvate). For an explanation, see the text.

In an attempt to simplify the data analysis, we divided the entire dataset into the four metabolic patterns defined in **Figure 3**∶1) **Pattern 1**: lactate normal (≤2.5 mmol/L) and LPR below 25, 2) **Pattern 2**: high lactate (>2.5 mmol/L) and a concurrent LPR ≤25, 3) **Pattern 3:** increased lactate (>2.5 mmol/L) and increased LPR (>25), 4) **Pattern 4:** normal lactate (≤2.5 mmol/L) and increased LPR (>25). The frequency of each pattern and the descriptive statistics are summarized in **Table 3**.

**Table 3:** Summary of metabolites for each metabolic pattern.

Metabolic pattern	Thresholds	Suggested terminology	N	%	Glucose (mmol/L)^a^	Pyruvate (mmol/L) a	Lactate (mmol/L) a	Lactate-pyruvate ratio a
**1**	L ≤2.5	Normal metabolism	1722	32.5	1.43 (0.05–7.1)	0.097 (0.01–0.236)	1.8 (0.1–2.5)	18.0 (2.0–25.0)
	LPR ≤25							
**2**	L >2.5	Aerobic hyperglycolysis	1274	24.0	2.27(0.11–11.6)	0.185 (0.10–0.643)	3.8 (2.6–12.0)	21.8 (10.8–25.0)
	LPR ≤25							
**3**	L >2.5	Anaerobic metabolism	1744	32.9	1.28 (0.05–6.5)	0.128 (0.01–0.455)	4.4 (2.6–12.0)	31.3 (25–545.5)
	LPR >25							
**4**	L ≤2.5	Low pyruvate	565	10.7	0.93 (0.05–7.1)	0.069 (0.01–0.098)	2.1 (0.4–2.5)	28.8 (25–195.3)
	LPR >25							
TOTAL			5305	100	1.42 (0.05–11.6)	0.117 (0.01–0.644)	2.8 (0.1–12.0)	23.9 (2.0–545.5)

L, lactate; LPR, lactate-to-pyruvate ratio; N, number of readings; %, percentage.

a Median (minimum-maximum).

Pattern 4 (“low pyruvate”) was observed the least frequently (10.7%) and was analyzed in depth because it challenged our understanding of energetic metabolism. The most striking difference in this pattern was that the median concentration of glucose in the brain ECF was significantly lower than the brain glucose in the other three groups (Kruskal Wallis, chi-squared = 471.4, df = 3, p<0.001). Of the 565 samples included in this profile (“low pyruvate”), we had good subcutaneous MD data for 435 of them (77%). In this subset, we defined a threshold of 3.6 mmol/L (65 mg/dL) to define low subcutaneous glucose and potential hypoglycemia. An excellent correlation has been shown in diabetic patients by continuous monitoring of glucose by vascular and subcutaneous MD [35]. Furthermore, in neurocritical patients, a good correlation has been shown between blood glucose and subcutaneous glucose six hours after insertion of the MD catheter [36]. Therefore, we considered subcutaneous MD glucose an acceptable surrogate for hypoglycemia. According to this threshold, only 69 subcutaneous samples (15.8%) showed a low subcutaneous glucose concentration.

## Discussion

Brain MD allow for continuous neurochemical monitoring and the unique opportunity to explore the disturbances in metabolism in neurocritical patients. However, MD is not yet a routine neuromonitoring tool and the interpretation of the results is still plagued by methodological problems, disagreement in reference ranges and thus in diagnostic thresholds, and uncertainties in the interpretation of the results. Our data showed poor agreement between the two most commonly used biomarkers for impaired energetic metabolism and highlighted the need for a common ground for future comparisons of results across studies.

### The ongoing problem of the reference ranges

One of the crucial steps for any biomarker is determining its reference range (i.e., its normal range) in a cohort of disease-free patients. For brain MD, this approach is limited by its invasiveness and by ethical issues. In MD, technical issues increase the complexity of determining appropriate reference intervals. The infusion flow rate, the length of the dialyzing membrane, and the fluid used as a perfusate modify the amount of the recovered analyte. The concentration of the analyte in the dialysate reflects a variable–and sometimes unknown–percentage of the real tissue concentration [37]. To minimize this problem, most centers use a standardized method with 10 mm length membranes, mock CSF as the perfusate, and a flow rate of 0.3 µL/min.

The most commonly used reference range was taken from the study of Reinstrup et al. conducted on nine awake and anesthetized patients operated on for posterior fossa tumors in whom a ventricular drainage was inserted [5]. In the awake patients, the mean lactate was 2.9±0.9 mmol/L and the LPR was 23±4. Applying the conventional rule–using 1.96 standard deviation– for calculating the upper thresholds, gives an upper threshold for lactate of 4.6 mmol/L and of 31 for the LPR. However, due to the small sample size, the anesthetic conditions –isoflurane was used in some of them– and the changes in perfusion speed, using the traditional method for calculating the upper and lower threshold from this cohort is misleading. In addition, volatile anesthetics may induce a significant increase in lactate due to reversible mitochondrial dysfunction [38], [39] and the awake patients were studied immediately after surgery and therefore metabolic stress or even hyperglycolysis could be a confounding factor.

Most papers in the last decade that studied patients with TBI and aneurysmatic subarachnoid hemorrhage used an upper threshold of 4.0 mmol/L, a threshold that Timofeev et al. showed was useful in predicting poor outcomes in TBI patients [7]. However, we believe that such a high threshold is difficult to justify, especially when much lower thresholds are used in blood or other organs [40], [41]. In our study, we used an upper threshold of 2.5 mmol/L for lactate corresponding to a real ECF lactate of around 3.7 mmol/L according to lactate’s relative recovery of 67% calculated by Hutchinson et al. using the same methodology as our study [42]. For the LPR, we selected the threshold of 25 used in most studies because this ratio is robust to changes in fluid recovery [2], [6]–[8], [26], [43].

### Lactate and the LP ratio need to be combined as screening tools

Moderate and severe TBIs cause frequent alterations in brain energy metabolism even in macroscopically normal brain tissue. At the predefined threshold of 2.5 mmol/L, we found an increase in brain lactate in 56.9% of the samples analyzed (**Table 2**). In 1496 samples (28.2%), lactate was above 4.0 mmol/L. Our data are in agreement with a recent study from our group in which the effects of normobaric hyperoxia on brain energy metabolism were evaluated and 45% of the patients had lactate levels above 3 mmol/L despite the probe placement being in macroscopically non-injured brain tissue and with a PtiO_2_ within the normal range in most of them [8]. If probes had been placed in traumatic penumbra and not in brain that appeared normal, the frequency of increased lactate could have been even higher. However, despite the high prevalence of elevated lactate, concurrent elevation of lactate and the LPR (>25) was observed in only 33% of the microdialysates, suggesting that a true anaerobic pattern was detected in one third of the samples.

We found a remarkable disagreement between lactate and the LP ratio in classifying the brain energy state in a simple dichotomized scale (normal versus abnormal/anaerobic). Methods used to evaluate inter-rater agreement showed a non-acceptable agreement between lactate and the LPR in classifying samples. Therefore, the conventional approach of using lactate or the LPR alone–at any threshold– needs to be abandoned for metabolic profiling both in clinical settings and for reporting clinical research. Any attempt to classify the metabolic status as normal or abnormal (i.e., “anaerobic”) using only lactate can lead to an overdiagnosis of anaerobic metabolism when not combined with pyruvate and the LPR. Hovda’s group has shown the major flaws of using this approach in different studies [2], [44].

The meaning of high lactate in the brain has been extrapolated from research in shock and sepsis and from experimental models of brain ischemia. An increase in lactate in any organ has been traditionally associated with low tissue perfusion and anaerobic metabolism [29]. In addition, hyperlactatemia has been consistently associated with an increase risk of death in patients with various types of shock [45]. However, in critical care research, pyruvate and consequently the LPR have been rarely used because of the complex handling of pyruvate that is required and the lack of analytical tools to assay it at the bedside [29]. The availability of portable MD bedside analyzers has changed this scenario, allowing the systematic use of pyruvate. The incorporation of pyruvate into neurocritical care has made it increasingly clear that the production and clearance of lactate in any tissue is multifactorial and far more complex than expected [2], [14]. In brief, lactate concentration is a highly sensitive indicator of upregulated glycolytic flux but pyruvate levels are necessary in order to differentiate whether the upregulation of the glycolytic flux is anaerobic (i.e., hypoxic) or simply an indicator of an increased use of the glycolysis under aerobic conditions.

### Metabolic patterns

We confirmed that some sort of metabolic impairment was present in 57% of the samples analyzed when we used a lactate threshold as a criterion. In the analysis of our data, we found it useful to partition the entire data set in the four patterns described in **Figure 3**. According to our data, only one third of the samples were compatible with a normal metabolism. An increase in ECF levels of lactate above 4.0 mmol/L and a high LPR (>40) have been consistently shown by independent groups to be highly sensitive predictors of poor outcomes and even of pronounced neural loss and brain atrophy [2], [7]. In an attempt to interpret the patterns we suggested the following working terminology: Normal metabolism for pattern 1, Aerobic hyperglycolysis for pattern 2, Anaerobic metabolism for pattern 3 and Low pyruvate for pattern 4. The frequency of each pattern and the descriptive statistics are summarized in **Table 3**. We believe this interpretation may be controversial and for a better understanding of these patterns there is need to combine MD data with brain oxygen monitoring data to verify or refute patterns 2 (“aerobic hyperglycolysis”) and 3 (“anaerobic metabolism”) and to prove whether our interpretation is reasonable.

Hypermetabolism was considered in the past a form of “hypoxia”. In a pivotal paper by Siggaard-Andersen et al., “hypermetabolic hypoxia” was defined as a situation in which ATP hydrolysis was not balanced by an increase in oxidative ATP synthesis. As a consequence, glycolysis was activated and lactate increased [46]. However, strictly speaking, we know that this pattern is present when oxygen delivery and utilization is within the normal range; therefore, this pattern has to be differentiated from any other form of tissue hypoxia. The hyperglycolytic pattern was defined in TBI by a pivotal study from Hovda’s group [2], [47] and emerges only when both lactate and the LP ratio are used. Bergsneider et al. showed hyperglycolysis in 56% of severe TBI patients studied using PET scanning within one week of injury and in patients with sufficient brain oxygen [47]. This pattern has also been reported in the brain of patients with aSAH [6], [26], [48]. In a pivotal paper, Hutchinson et al. in 17 patients with severe TBI used both MD and positron emission tomography (PET) parameters– using the glucose analogue [F^18^]-fluorodeoxyglucose (FDG) – to correlate the extracelullar levels of glucose, lactate and pyruvate with the regional cerebral metabolic rate for glucose (CMRglc) in the same region of interest [49]. The most relevant finding of this study is that there was a linear relationship between CMRglc and the MD levels of lactate and pyruvate but not with the LPR [49]. These data confirm the general view that the increase in CMRglc in these patients is often indicative of hyperglycolysis and not of a shift towards anaerobic metabolism.

The main difference between hyperglycolysis and an anaerobic profile is that the LPR is significantly increased in the latter. It has been postulated that glycolysis upregulation in an injured brain indicates a hypermetabolic state directed toward restoring perturbed ionic homeostasis or the reuptake of high extracellular levels of glutamate. Hypermetabolism following TBI occurs because oxidative phosphorylation normally runs at near maximal capacity and, consequently, an increased energy demand should be supplied by and increased in glycolysis [26]. Different authors have shown that a hyperglycolytic pattern is a predictor of good neurological outcomes in patients with aSAH compared with patients with an anaerobic pattern [6], [26].

The anaerobic pattern (high lactate/high LP ratio) was observed in one third of the analyzed samples. This pattern is well consolidated and it has been the only pattern detected in studies that have used only lactate for profiling energy metabolism. The LPR reflects the equilibrium between product and substrate of the reaction catalyzed by lactate dehydrogenase and the LPR is a good surrogate for the cytosolic oxido-reduction status [50]. In patients with aSAH, an anaerobic pattern is related to poor neurological outcomes [6], [26]. Cesarini et al. showed that when this pattern is associated with ischemia in a sample of patients with aSAH, it is also linked to concurrent low brain glucose concentrations [26]. However, although reduced ECF concentration of glucose is a hallmark of hypoxic ischemia, its concentration may be normal (or even high) in non-ischemic forms of hypoxia (anemia, hypoxemia, high-affinity hypoxia, etc.). This pattern is the most important in clinical settings; when detected, it requires a systematic approach to rule out all the classes of brain hypoxia.

The low pyruvate pattern detected in 10.7% of our samples was quite unexpected, given that this profile was already described by Hlatky et al. and confirmed by others [24], [44], [49], [51]. Hillered et al. called this pattern a type 2 elevation of the LP ratio [44], [51]. This pattern, in which lactate is within the normal range, is misleading and, if not detected, can be easily considered an indicator of anaerobic metabolism and thus indicative of brain hypoxia when it is not. In our series, this pattern was associated with a low brain ECF glucose concentration, as was described by Marcoux et al. [44] Because the brain is an obligate glucose consumer, its fuel depends on the glucose plasma levels. With all the limitations of our study (lack of matched oxygen data for this cohort), a low ECF glucose was associated with the drop in pyruvate concentrations and the increase in the LP ratio. However, hypoglycemia was infrequent. In all the samples in which we had good quality subcutaneous data, only 16% presented a low subcutaneous concentration of glucose; hypoglycemia might therefore have been the cause. Marcoux et al. suggested that a low pyruvate and a high LPR may be secondary to mitochondrial dysfunction [44]. However, when mitochondrial dysfunction has been reproduced in experimental models, a high lactate level, together with a high LPR has been consistently found [39], [52]. A third alternative to explain this pattern and the concomitant low levels of glucose is the shunting of glucose-6-phosphate toward alternative metabolic pathways, specifically the pentose phosphate pathway. This process has been observed in both experimental models and in clinical studies of TBI [53], [54]. Hutchinson et al. in their combined MD/PET-FDG study suggested that the low levels of glucose in the ECF of head-injured patients are a consequence of an increase in substrate demand rather than inadequate substrate delivery [49]. However, a better understanding of this pattern requires additional studies with simultaneous measurements of plasmatic glucose, lactate, pyruvate, and brain tissue oxygen. When mitochondrial dysfunction is present, as hypothethised by some, the tissue oxygen is not used and the amount of dissolved oxygen is significantly increased [39], [52].

### Metabolic impairment and brain hypoxia

Evidence accumulated in the last two decades shows that non-ischemic causes of brain hypoxia are frequent. The most comprehensive classification of tissue hypoxia was developed by Siggaard-Andersen et al. in 1995 [46], [55]. However, so far, this classification has been rarely used in neurocritical care. The brain is a highly aerobic organ that requires a sufficient supply of oxygen (O_2_) to the mitochondria to maintain adequate ATP production. The supply of O_2_ to the brain is multifactorial and depends on cerebral blood flow (CBF), the ability of the blood to transport O_2_, hemoglobin (Hb) oxygen affinity, Hb characteristics, O_2_ diffusive conductance from arterial capillaries to the cells, and the arterial oxygen pressure gradient between the capillaries and the intracellular compartment [56]. It is thus obvious that the potential causes of hypoxia are multiple and not limited to ischemia (i.e., ischemic hypoxia), a term that should be reserved to describe brain hypoxia caused by a reduction in CBF uncoupled to brain metabolism. In a recent brain MD study conducted by Nelson et al. on TBI patients, highly impaired energy metabolism was very prevalent [57]. However, the relationships between MD and either intracranial pressure and/or cerebral perfusion pressure were very weak and did not explain the observed energetic disturbances. These findings suggest that other factors besides pressure and/or flow may be the main cause of metabolic perturbations in these patients [57].

The discussion about lactate has been plagued by the same problems as that of hypoxia. For many years, the primary causes of lactate production by any tissue were thought to be either low levels of blood flow (ischemia) or low levels of blood oxygen content (hypoxemia). However, this oversimplification is misleading. In the Siggaard-Andersen classification, nine types of tissue hypoxia were described. If “hypermetabolic hypoxia” is excluded and the uncoupling hypoxia is merged with hystotoxic hypoxia (i.e., mitochondrial dysfunction), seven profiles remain that are very useful as a theoretical framework [46], [55]. In the original Siggaard-Andersen classification, histotoxic hypoxia is a term that is equivalent to mitochondrial dysfunction and reflects the situation in which oxygen delivery is sufficient but the respiratory chain cannot utilize it. This situation stimulates glycolysis but without a decrease in pyruvate. There is increasing experimental and indirect clinical evidence suggesting that severe TBI may be associated with mitochondrial dysfunction. In the presence of adequate oxygenation, the only available BMs that indicate mitochondrial dysfunction are lactate and the LP ratio. In the last decade, different groups have provided important experimental data that point to mitochondria as a cause of many metabolic disturbances and therefore the potential therapeutic target [58], [59].

### Study limitations

Because the main goal of our study was to evaluate the frequency and agreement between lactate and the LPR, hourly data were pooled from the entire cohort of patients. Therefore, we cannot analyze the influence of the metabolic profile in the clinical evolution and short- or long-term patient’s outcome and caution needs to be exercised in the interpretation of our findings. In addition, our study focused on the metabolic disturbances found in brain tissue that appeared normal. Therefore, although this sample is probably representative of most of the non-injured brain, further studies need to be conducted to clarify whether brain metabolism in such areas is representative of the energetic metabolism in the whole brain or at least of the hemisphere where the probe is implanted.

A third limitation is that patients may show different patterns at different time points after injury. This fact was not explored in our study, nor was the coexistence of different metabolic patterns in the same patient. An additional limitation is that our study did not consider the potential causes of specific profiles, nor we did correlate them with the more common variables that are routinely monitored in TBI patients (intracranial pressure, cerebral perfusion pressure, brain oxygenation, etc.).

## Conclusions and Future Directions

Brain MD allows for the screening of disorders in energy metabolism in patients with acute brain injuries. Our study showed that metabolic abnormalities are frequent in the macroscopically normal brain of patients in the acute phase of TBI. These disorders by their nature are clinically silent until their late stages, at which point patients may suffer significant irreversible brain damage; early detection is important. A very poor agreement between lactate and the LPR was found when classifying normal or abnormal metabolism. Our data suggest that the concentration of lactate in MD should always be interpreted taking into consideration the LPR to distinguish between anaerobic metabolism and hypermetabolism. The use of both lactate and the LPR can be used at the bedside to classify the metabolic profiles in four patterns that can be useful for further exploring the characteristics, their causes, and their prognostic values. Whether or not a normal metabolic pattern has any influence in functional outcome needs to be explored in further studies with a different design and a bigger sample size in which summary measures –the area under the curve above certain thresholds, the percentage of time above a threshold, etc.– are incorporated.

The pattern of low pyruvate was associated with a low concentration of ECF glucose but not hypoglycemia. This pattern and its clinical significance need further clarification, especially because even in situations of normoglycemia the transport of glucose across the blood-brain barrier might be impaired in these patients. Despite the discussed limitations, we believe that the classification provided here can be a starting point for further research in the impairment of energetic metabolism. The definition and better characterization of different metabolic patterns provides an opportunity to study their causes, modulate them, and potentially influence the outcomes of patients with acute brain injuries.
