# Correction: Lactate and the Lactate-to-Pyruvate Molar Ratio Cannot Be Used as Independent Biomarkers for Monitoring Brain Energetic Metabolism: A Microdialysis Study in Patients with Traumatic Brain Injuries

**DOI:** 10.1371/journal.pone.0111821

**Published:** 2014-10-22

**Authors:** The PLOS ONE Staff

For the sake of clarity, the authors have provided the tables of their article in the format given prior to our typesetting process. Please download this supporting Table S1 to view the tables of this manuscript in the layout the authors intended.

## Supporting Information

Table S1 Original format for Table 1, Table 2, and Table 3 of “Lactate and the Lactate-to-Pyruvate Molar Ratio Cannot Be Used as Independent Biomarkers for Monitoring Brain Energetic Metabolism: A Microdialysis Study in Patients with Traumatic Brain Injuries”
(PDF)
