# Supplementary material for: Correction: Lactate and the Lactate-to-Pyruvate Molar Ratio Cannot Be Used as Independent Biomarkers for Monitoring Brain Energetic Metabolism: A Microdialysis Study in Patients with Traumatic Brain Injuries
Source: PLoS One. 2014 Oct 22;9(10):e111821. doi: 10.1371/journal.pone.0111821 (PMC4206457; doi:10.1371/journal.pone.0111821)
Supplement: Table S1 — Original format for Table 1, Table 2, and Table 3 of “Lactate and the Lactate-to-Pyruvate Molar Ratio Cannot Be Used as Independent Biomarkers for Monitoring Brain Energetic Metabolism: A Microdialysis Study in Patients with Traumatic Brain Injuries” (PDF) [file pone.0111821.s001.pdf]

Table 1. Demographic and clinical characteristics of the patients

Sex[a]	
Man	36 (78%)
Woman	10 (22%)
Age[b]	35 (±15)
Initial GCS[c]	6 (5,8)
Initial CT classification[a]	
II	26 (56.5%)
III	5 (10.9%)
IV	5 (10.9%)
V	8 (17.4%)
VI	2 (4.3%)
GOSE (6 months)[a]	
Good outcome	15 (32.6%)
Bad outcome[d]	18 (39.1%)
Dead[e]	8 (17.4%)
Lost to 6-months follow-up	5 (10.9%)

GOSE, Extended Glasgow Outcome Scale; GCS, Glasgow Coma Scale
[a] Number of cases (percentage)
[b] Mean (standard deviation)
[c] Median (first quartile, third quartile)
[d] Patients with upper or lower severe disability or vegetative state
[e] Mortality at hospital discharge

Table 2. Intermethod agreement in the classification of dialysate samples by lactate and the LPR

			Lactate		
			Normal metabolism *L ≤ 2.5*	***Abnormal metabolism*** *L > 2.5*	***Total***
Lactate-to-pyruvate ratio	***Normal metabolism***	***N***	1722	1274	2996
	LP ratio ≤ 25	***%***	32.5%	24.0%	56.5%
	Abnormal metabolism	***N***	565	1744	2309
	LP ratio > 25	***%***	10.7%	32.9%	43.5%
	TOTAL	***N***	2287	3018	**5305**
		%	43.1%	56.9%	**100%**

LP, lactate-to-pyruvate; L, lactate; >, greater than; ≤, less than or equal to; N, number of readings; %, percentage.

Table 3. Summary of metabolites for each metabolic pattern

Metabolic pattern	Thresholds	Suggested terminology	N	%	Glucose (mmol/L)[a]	Pyruvate (mmol/L)[a]	Lactate (mmol/L)[a]	Lactate-pyruvate ratio [illegible]
**1**	L ≤ 2.5<br>LPR ≤ 25	Normal metabolism	1722	32.5	1.43 (0.05-7.1)	0.097 (0.01-0.236)	1.8 (0.1-2.5)	18.0 (2.0-25.0)
**2**	L > 2.5<br>LPR ≤ 25	Aerobic hyperglycolysis	1274	24.0	2.27(0.11-11.6)	0.185 (0.10-0.643)	3.8 (2.6-12.0)	21.8 (10.8-25.0
**3**	L > 2.5<br>LPR > 25	Anaerobic metabolism	1744	32.9	1.28 (0.05-6.5)	0.128 (0.01-0.455)	4.4 (2.6-12.0)	31.3 (25-545.5)
**4**	L ≤ 2.5<br>LPR > 25	Low pyruvate	565	10.7	0.93 (0.05-7.1)	0.069 (0.01-0.098)	2.1 (0.4-2.5)	28.8 (25-195.3)
TOTAL			5305	100	1.42 (0.05-11.6)	0.117 (0.01-0.644)	2.8 (0.1-12.0)	23.9 (2.0-545.5

L, lactate; LPR, lactate-to-pyruvate ratio; N, number of readings; %, percentage.
a Median (minimum-maximum).
